# Cure of Micrometastatic B-Cell Lymphoma in a SCID Mouse Model Using ^213^Bi-Anti-CD20 Monoclonal Antibody

**DOI:** 10.2967/jnumed.122.263962

**Published:** 2023-01

**Authors:** Gregory T. Havlena, Nirav S. Kapadia, Peng Huang, Hong Song, James Engles, Martin Brechbiel, George Sgouros, Richard L. Wahl

**Affiliations:** 1 Kaiser Permanente Radiology, Fontana, California;; 2 Dartmouth–Hitchcock Medical Center, Lebanon, New Hampshire;; 3 Johns Hopkins University School of Medicine, Baltimore, Maryland;; 4 Section of Nuclear Medicine, Stanford University School of Medicine, Stanford, California;; 5 National Institutes of Health, Bethesda, Maryland; and; 6 Mallinckrodt Institute of Radiology, Washington University in St. Louis School of Medicine, St. Louis, Missouri

**Keywords:** ^213^Bi, alpha emitter, B-cell non-Hodgkin lymphoma, animal model, bioluminescence, radioimmunotherapy

## Abstract

We studied the feasibility of using the α-emitting ^213^Bi-anti-CD20 therapy with direct bioluminescent tracking of micrometastatic human B-cell lymphoma in a SCID mouse model. **Methods:** A highly lethal SCID mouse model of minimal-tumor-burden disseminated non-Hodgkin lymphoma (NHL) was established using human Raji lymphoma cells transfected to express the luciferase reporter. In vitro and in vivo radioimmunotherapy experiments were conducted. Single- and multiple-dose regimens were explored, and results with ^213^Bi-rituximab were compared with various controls, including no treatment, free ^213^Bi radiometal, unlabeled rituximab, and ^213^Bi-labeled anti-HER2/*neu* (non–CD20-specific antibody). ^213^Bi-rituximab was also compared in vivo with the low-energy β-emitter ^131^I-tositumomab and the high-energy β-emitter ^90^Y-rituximab. **Results:** In vitro studies showed dose-dependent target-specific killing of lymphoma cells with ^213^Bi-rituximab. Multiple in vivo studies showed significant and specific tumor growth delays with ^213^Bi-rituximab versus free ^213^Bi, ^213^Bi-labeled control antibody, or unlabeled rituximab. Redosing of ^213^Bi-rituximab was more effective than single dosing. With a single dose of therapy given 4 d after intravenous tumor inoculation, disease in all untreated controls, and in all mice in the 925-kBq ^90^Y-rituximab group, progressed. With 3,700 kBq of ^213^Bi-rituximab, 75% of the mice survived and all but 1 survivor was cured. With 2,035 kBq of ^131^I-tositumomab, 75% of the mice were tumor-free by bioluminescent imaging and 62.5% survived. **Conclusion:** Cure of micrometastatic NHL is achieved in most animals treated 4 d after intravenous tumor inoculation using either ^213^Bi-rituximab or ^131^I-tositumomab, in contrast to the lack of cures with unlabeled rituximab or ^90^Y-rituximab or if there was a high tumor burden before radioimmunotherapy. α-emitter–labeled anti-CD20 antibodies are promising therapeutics for NHL, although a longer-lived α-emitter may be of greater efficacy.

## Visual Abstract

**Figure:**
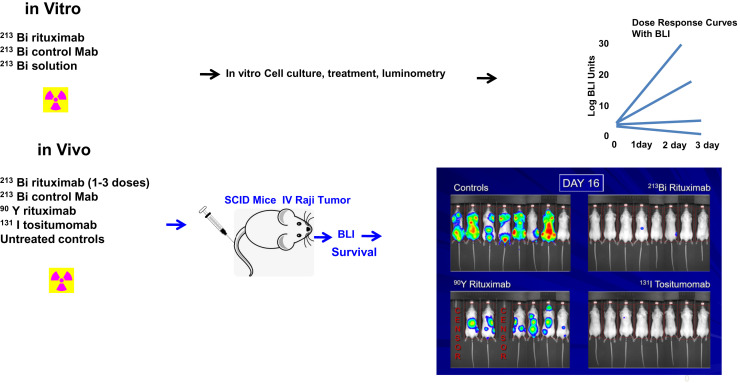

Interest in radioimmunotherapy began last century but heightened with the Food and Drug Administration (FDA) approval in 2002 and 2003 of the anti-CD20 radioimmunotherapies ^90^Y-ibritumomab tiuxetan (Zevalin; Acrotech Biopharma) and tositumomab and ^131^I-tositumomab (Bexxar; GlaxoSmithKline). They represent the only FDA-approved radioimmunotherapy agents (*1*), though ^131^I-tositumomab is no longer marketed in the United States and use of ^90^Y-ibritumomab tiuxetan is limited (*2*).

The FDA-approved radionuclides for anti-CD20 radioimmunotherapy emit β-particles, which differ from α-particle or Auger-electron radiation (*3**,**4*). β-particles deposit energy along a relatively large distance (pathlength), resulting in a relatively low energy deposition per unit distance traveled (linear energy transfer). The β-particle emitted by ^90^Y travels an average pathlength of 2.7 mm, with an average decay emission energy of 0.93 MeV and an average energy transfer of 0.93 MeV/2.7 mm (≈0.34 keV/μm). The ^131^I β-particle travels an average distance of 0.8 mm as it deposits an average of 0.19 MeV (*5*).

Although monoclonal antibodies are targeted to individual cancer cells, ^90^Y and ^131^I deposit much of their energy beyond the single-tumor-cell diameter. The long β-particle paths for ^131^I and ^90^Y are potentially advantageous if all the targeted tumor cells do not bind the antibody or if delivery of the antibody is heterogeneous in tumors, as it results in a more uniform radiation dose across the tumor. However, the rather long pathlength makes it more difficult for these agents to kill isolated single neoplastic cells or very small oligometastatic tumors, as much of the energy is deposited remotely from the tumor. Furthermore, the relatively long pathlengths traveled by these decay particles can increase the likelihood of normal-tissue toxicity (*6*). α-particles are helium nuclei that travel a shorter pathlength and deposit approximately 200–400 times more energy along their path distance. Notably, the α-particle emitted by ^213^Po (the short-lived daughter species of ^213^Bi) travels an average of only 0.08 mm and is strikingly more energetic than its β-particle–emitting counterparts (8.35 MeV) (*5*). In the case of radioimmunotherapy, this translates into more frequent double-stranded DNA breaks (*7*) and an increased likelihood of caspase-mediated apoptosis (*8*). ^213^Bi and other α-emitters represent promising candidates for the single-cell kill necessary to cure isolated cancer cells or very small volumes of metastatic disease. There is now an FDA-approved α-emitting radiopharmaceutical for bone metastases, ^223^Ra-dichloride (*9*).

The experiments we conducted used a bioluminescent imaging (BLI) reporter system for real-time monitoring of treatment response, affording a sensitive view of in vivo tumor kill kinetics in a very low tumor burden (*10**,**11*).

We describe the synthesis and evaluation of the monoclonal anti-CD20 antibody rituximab, labeled with the α-emitter ^213^Bi (half-life [t½], 46 min). In vitro response to treatment was monitored with serial luminometry against appropriate controls, whereas the in vivo response was monitored via sequential optical imaging of the disseminated Raji (non-Hodgkin) lymphoma cell tumor burden in SCID mice. ^213^Bi-rituximab has been used in vitro and has shown substantial antitumor activity. It also has been used for biodistribution studies in animal models of human non-Hodgkin lymphoma (NHL). There are preliminary data on the use of ^213^Bi-rituximab in the treatment of patients with NHL. However, evaluation as an in vivo therapeutic has not been extensive, presumably in part because the short t½ of ^213^Bi perhaps seems ill-matched to the relatively slow targeting of intact monoclonal antibodies to solid tumors (*12**,**13*). In pretargeting settings, ^213^Bi has shown promise to treat subcutaneous and disseminated NHL (*12*). However, our evaluation focused on an intravenously delivered and widely disseminated tumor model in which a ^213^Bi-labeled intact radioantibody might be expected to target to tumor far more rapidly than delivery of such a large, intact antibody to a relatively less well-perfused subcutaneous tumor. We also performed studies comparing ^213^Bi-rituximab with the previously FDA-approved ^131^I-anti-CD20 (tositumomab) and ^90^Y-rituximab.

## MATERIALS AND METHODS

Detailed methods appear in the supplemental materials (available at http://jnm.snmjournals.org). Epstein-Barr virus–positive human Raji lymphoma tumor cells were lentivirally transfected with green fluorescent protein and luciferase reporters of gene expression (*14*). Rituximab (mouse–human chimeric IgG monoclonal anti-CD20) was obtained from Genentech/Biogen Idec, and murine anti-HER-2/*neu* antibody 7.16.4 was obtained from the Sgouros laboratory. Antibody integrity was verified via sodium dodecyl sulfate polyacrylamide gel electrophoresis. Raji cell surface expression of CD20 and antibody immunoreactivity were verified by a quantitative CD20 assay. ^131^I-tositumomab was obtained from the Johns Hopkins Outpatient Center.

Rituximab and anti-HER-2/*neu* were conjugated to SCN-CHX-A″-DTPA as previously described (*15**,**16*). The average number of chelates per antibody was approximately 1.6 (*17*). Immunoreactivity was determined by the method of Lindmo et al. (*18*).

^225^Ac was purchased from Oak Ridge National Laboratory or Curative Technologies. ^213^Bi was eluted from an ^225^Ac generator (*19*). Rituximab or antibody 7.16.4 conjugated to the chelate was prepared. The reaction efficiency and purity of radioimmunoconjugates were determined with thin-layer chromatography. ^90^Y-labeled rituximab was similarly prepared.

After the generation of standard curves for Raji–green fluorescent protein–luciferase cell bioluminescence, 5 × 10^4^ cells were measured on a Monolight 3010 luminometer (Becton Dickinson). Samples were divided into 4 groups: untreated controls, free radionuclide, and ^213^Bi-radiolabeled anti-CD20 either blocked or unblocked with unlabeled anti-CD20. Antigenic blockade was accomplished with a 24-h predose of unlabeled anti-CD20 at a concentration of 50 μg/mL. Serial counts were obtained daily for 7 d or until no viable cells remained in culture in quadruplicate experiments.

CB57 (CB17) BALB/c SCID mice (female) were intravenously injected with either 5.0 × 10^5^ or 1.0 × 10^6^ Raji–green fluorescent protein–luciferase lymphoma cells on day 0. Immediate in vivo BLI confirmed successful intravenous tumor injection by the presence of quantifiable signal within the lungs. In the confirmed absence of tumor, mice were once again inoculated with intravenous tumor and reimaged to confirm tumor dissemination.

In each of the 4 in vivo experiments, mice inoculated with tumor cells were treated with ^213^Bi-rituximab or one of several controls (Table 1). Single- and multiple-dose regimens were explored and compared with no treatment, free ^213^Bi radiometal, mass-equivalent doses of unlabeled rituximab, and ^213^Bi-labeled anti-HER2/neu (nonspecific antibody). ^213^Bi-rituximab was also compared with anti-CD20 antibodies radiolabeled with β-emitters, ^131^I-tositumomab, and ^90^Y-rituximab. In all except one of the experiments, treatments were initiated once exponential growth in tumor signal was established (*14*) to have taken place by day 7 after tumor inoculation. Treatment was initiated at an earlier time point (day 4) in a study comparing ^213^Bi-rituximab with ^131^I-tositumomab or ^90^Y-rituximab. Radioactivity was measured in a Capintec dose calibrator (*20*).

**TABLE 1.** Summary of Mouse Experiments

Treatment	Cells injected	Cures (*n*)
Group 1	1 × 10^6^	0/5
Untreated		
^ 213^Bi-rituximab, 925 kBq (day 7)	1 × 10^6^	0/5
^ 213^Bi-rituximab, 3,700 kBq (day 7)	1 × 10^6^	0/5
^ 213^Bi, free, 1,295 kBq (day 7)	1 × 10^6^	0/5
^ 213^Bi-HER2/neu, 1,295 kBq (day 7)	1 × 10^6^	0/5
10 μg of rituximab (day7)	1 × 10^6^	0/5
Group 2	5 × 10^5^	0/6
Untreated		
^ 213^Bi-rituximab, 2,775 kBq (day 7)	5 × 10^5^	2/6
^ 213^Bi-rituximab, 2,775 kBq (days 7, 13)	5 × 10^5^	3/7
10 μg of rituximab (days 7, 13)	5 × 10^5^	1/4
^ 213^Bi-HER2/neu, 2,775 kBq (day 7)	5 × 10^5^	0/5
Group 3	1 × 10^6^	0/6
Untreated		
^ 213^Bi-rituximab, 2,775 kBq (day 7)	1 × 10^6^	0/6
^ 213^Bi-rituximab, 2,775 kBq (days 7, 12)	1 × 10^6^	0/6
^ 213^Bi-rituximab, 2,775 kBq (days 7, 12, 19)	1 × 10^6^	0/6
Group 4	1 × 10^6^	0/8
Untreated		
^ 213^Bi-rituximab, 3,700 kBq (day 4)	1 × 10^6^	6/8
^ 131^I-tositumomab, 2,035 kBq (day 4)	1 × 10^6^	6/8
^ 90^Y-rituximab, 925 kBq (day 4)	1 × 10^6^	0/8
10 μg of rituximab (day 4)	1 × 10^6^	1/8

Tumor burden was followed using the Xenogen IVIS 200 series imaging system (PerkinElmer) every 2–7 d. Each animal served as its own reference, with results normalized to baseline pretreatment tumor burdens obtained on day 4 or 6. As tumor burden increased, the necessary acquisition time decreased. Optical images were analyzed via software provided by the manufacturer. Rectangular regions of interest were drawn around individual mice, and average radiance [photons/(s)(cm^2^)(sr)] was calculated by the software. This is also referred to as the relative light units per minute. Mice were followed with intermittent optical imaging for 28, 131, 49, and 85 d in in vivo experiments 1, 2, 3, and 4, respectively. In accordance with ethical guidelines, monitoring of a specific animal was discontinued if it died or showed evidence of hind-leg paralysis (HLP) warranting euthanasia. In the first of 4 in vivo experiments, animals were imaged for a preset period of 4 wk, at which time all surviving animals in the study were killed for pathologic analysis. In the remaining 3 studies, intermittent imaging was continued either until all mice in a given study were deceased (experiment 3) or until all mice in the study with any evidence of tumor progression had died (experiments 2 and 4). In the latter category, mice without evidence of tumor progression were imaged for an additional several weeks after the final tumor-related death in the study to ensure that they did not develop bioluminescent signal in excess of baseline as evidence of tumor progression. In all except experiment 1, overall survival was assessed by monitoring the mice after the imaging period had ended until death or hind limb paralysis developed or when the remaining animals needed to be killed for logistical reasons.

In each study, the time from treatment until death (or hind limb paralysis) was assessed by Kaplan–Meier analysis, ANOVA, and *t* tests. The relative light units were monitored for each animal before and at multiple time points after therapy. A log transformation was applied to normalized relative light units to analyze data from day 6 onward. A mixed-effects model was fitted for each group separately to estimate its normalized relative-light-unit growth rate after day 6 (the baseline day). Calculations were performed using SAS software.

Representative animals were identified for pathologic assessment at the conclusion of experiment 1, when their disease had progressed sufficiently that humane euthanasia was required and when the studies were terminated with no evidence of tumor progression in experiments 2–4.

## RESULTS

The CHX-A″ DTPA-conjugated antibodies were eluted from the reaction solution and concentrated to achieve final concentrations of approximately 10 mg/mL, with an average of 1.6 chelators per antibody. After radiolabeling and purification, at least 98.0% purity was achieved. Lineweaver–Burk extrapolations generally determined the immunoreactive fraction to be at least 50% for the anti-CD20 constructs.

**FIGURE 1.**
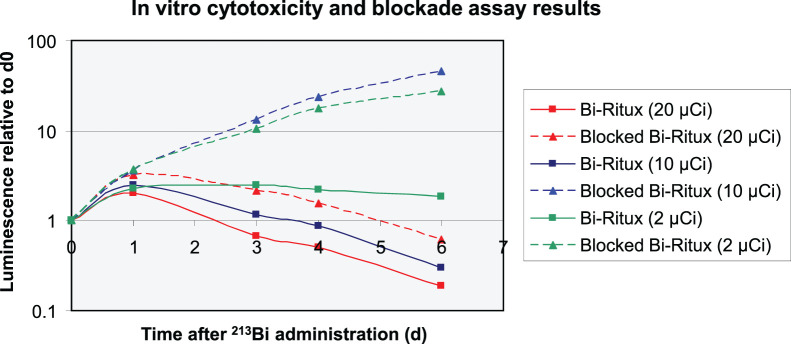
In vitro cytotoxicity by luminometry. Antigenic blockade was performed with 50 μg/mL application of unlabeled rituximab for 24 h before dosing with varying amounts of activity. Dose-dependent ^213^Bi-rituximab cell kill is substantially blocked with cold rituximab. *x*-axis represents days of assessment (0–6 d). Controls (not shown), antigen (CD-20) blocked ^213^Bi-rituximab (74 and 370 kBq did not vary from one another over 6 d, *P* = not statistically significant). Two, 10, and 740 kBq of ^213^Bi-rituximab and 740 kBq of ^213^Bi had significant antitumor effects vs. control and 74- and 370-kBq ^213^Bi-rituximab–blocked groups (*P* < 0.01). ^213^Bi-rituximab and free ^213^Bi at 740-kBq dose were comparable (*P* = not statistically significant). 1 μCi = 37 kBq.

Free ^213^Bi at doses of at least 370 kBq/mL had dose-dependent antitumor effects in vitro (*P* < 0.05). A 74 kBq/mL dose of ^213^Bi-rituximab demonstrated selective cytotoxic effects (*P* < 0.01) (Fig. 1). Specific cytotoxicity was absent (*P* = not statistically significant) with rituximab blockade. Among unblocked samples, the number of cells doubled over a 6-d period, whereas cells treated with the same dose after antigenic blockade multiplied 27-fold (*P* < 0.01). At the 370 kBq/mL dose of ^213^Bi-rituximab, net cytotoxicity (fewer cells than baseline) occurred within 4 d (*P* < 0.01). This effect was blocked by rituximab (*P* < 0.01). Complete cytotoxicity was observed at a 740 kBq/mL dose with or without antigenic blockade (*P* = 0.48) (Fig. 1). There was no difference in cell survival between the untreated controls and samples treated with 74 kBq/mL of free ^213^Bi, but free ^213^Bi and 370 and 740 kBq had antitumor effects (*P* = not statistically significant; Supplemental Fig. 1).

Table 1 summarizes the 4 in vivo experimental studies. Successful tumor injection was confirmed with immediate in vivo BLI. The absence of quantifiable signal above baseline, requiring repeat tumor injection, occurred in approximately 5% of injections. All groups had quantifiable tumor bioluminescence detectable on the day of tumor inoculation. No differences in absolute tumor signal were found between any of the groups before the day of treatment in any of the 4 in vivo experiments (*P* = not statistically significant). All groups had tumor growth above baseline on the last pretreatment day. Initially intense lung cellular accumulation visible by BLI cleared quickly, so that 2 d after tumor administration, tumor burden was undetectable. Tumor was reliably detected sparsely throughout the animal 4 d after injection. Three mice had a markedly increased tumor burden compared with the others (*P* < 0.01) and were excluded from the therapeutic study because they developed macroscopic disease.

**FIGURE 2.**
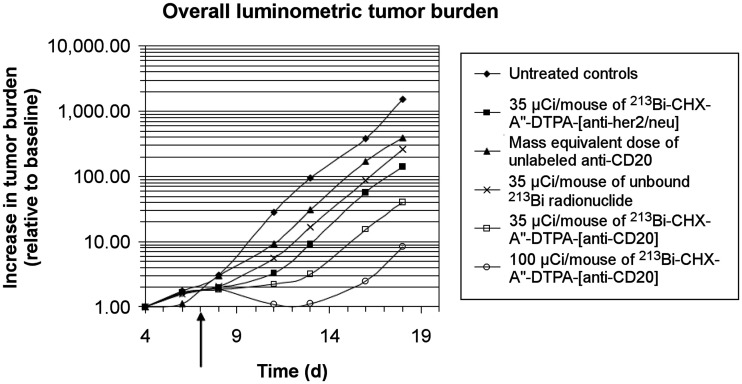
Experiment 1. In vivo tumor BLI growth curves are shown for various treatment groups. Results are normalized to baseline tumor burdens on day 4 (log scale). Time is measured from point of intravenous tumor inoculation. Statistical significance between various treatment and control arms at day 18 are displayed in Supplemental Table 1. 1 μCi = 37 kBq.

The overall survival duration was not assessed in experiment 1 because the animals were intentionally killed 28 d from tumor injection. After 28 d, all untreated mice had died or developed HLP (requiring euthanasia), whereas in the groups treated with a single dose of either 1,295 kBq or 3,700 kBq of ^213^Bi-rituximab, all mice were alive without HLP. Two mice (40%) remained alive in the unlabeled rituximab group, and 3 mice (60%) were alive in each of the other control groups (1,295 kBq of ^213^Bi free radiometal and 1,295 kBq of ^213^Bi-anti-HER2/*neu*; Fig. 2; Supplemental Fig. 2). Absolute tumor burdens with the varying therapies are shown in Supplemental Table 1.

**FIGURE 3.**
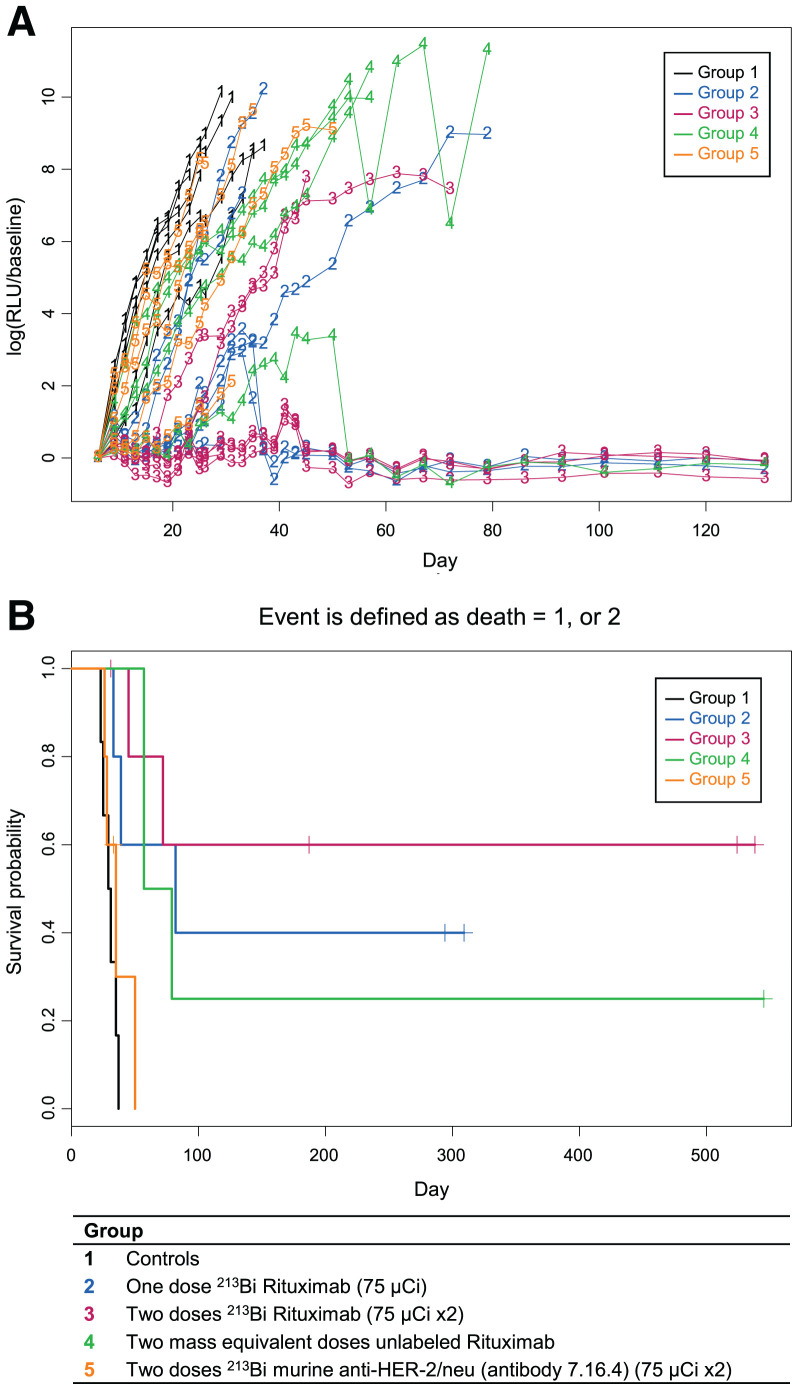
(A) Individual-animal relative light units show that 2 doses of ^213^Bi-rituximab were most effective. (B) Kaplan–Meier plots show prolonged survival of 2 doses and 1 dose of ^213^Bi-rituximab groups (groups 1 and 2). 1 μCi = 37 kBq; RLU = relative light unit.

In experiment 2, all untreated controls developed progressive tumor, with HLP occurring from days 23 to37 (Fig. 3). In the group treated with a single dose of 2,775 kBq of ^213^Bi-rituximab, only 50% of the mice progressed, and these died or developed HLP on days 33, 39, and 82. In the group treated with 2 doses of 2,775 kBq of ^213^Bi-rituximab, only 33% of the mice had disease progression, developing HLP on days 45 and 72. Seventy-five percent of mice treated with 2 mass-equivalent doses of unlabeled rituximab showed disease progression, developing HLP on days 57–79. All mice in the group receiving 2,775 kBq of ^213^Bi-anti-HER2/*neu* had disease progression and died or developed HLP from days 26 to 50.

**FIGURE 4.**
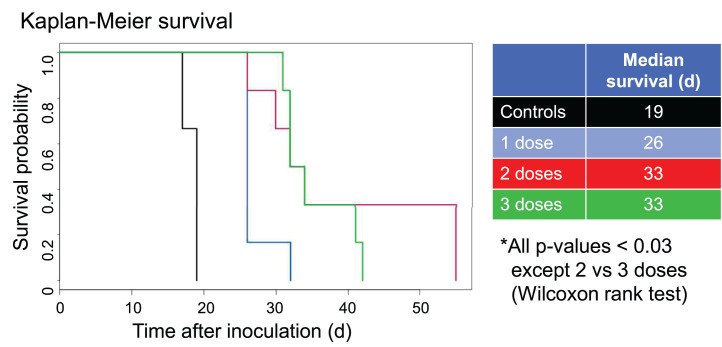
Experiment 3. Significant increase in median survival is seen with 1 dose of ^213^Bi-rituximab relative to controls and with 2 or 3 doses relative to 1 dose. This study used higher initial tumor inoculation with same timing of first treatment as in study 1. There were no cures in this study.

In experiment 3 (Fig. 4; Supplemental Fig. 3), all untreated controls developed HLP from days 17 to 19. Animals treated with a single dose of 2,775 kBq of ^213^Bi-rituximab developed HLP from days 26 to 32. Groups treated with 2 or 3 doses of 2,775 kBq of ^213^Bi-rituximab died or developed HLP from days 26 to 55 or days 31 to 42, respectively.

**FIGURE 5.**
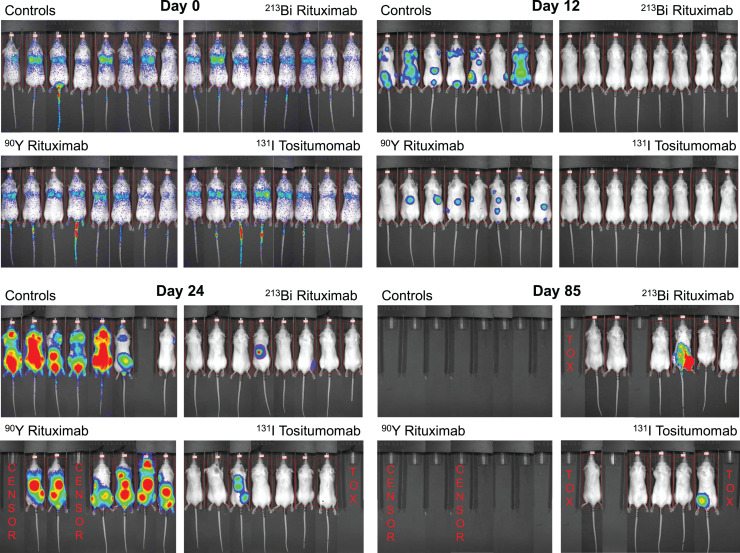
Selected bioluminescence images at varying times after treatment in experiment 4. Single treatment was given 4 d after injection of 10^6^ tumor cells intravenously (8 animals per group). BLI and survival were assessed. Treatment groups included controls (no treatment), 10 μg of unlabeled rituximab, 3,700 kBq of ^213^Bi-rituximab, 2,035 kBq of ^131^I-tositumomab, and 1,295 kBq of ^90^Y-rituximab. Survival is prolonged in ^213^Bi and ^131^I anti-CD20 groups vs. controls and ^90^Y-rituximab.

**FIGURE 6.**
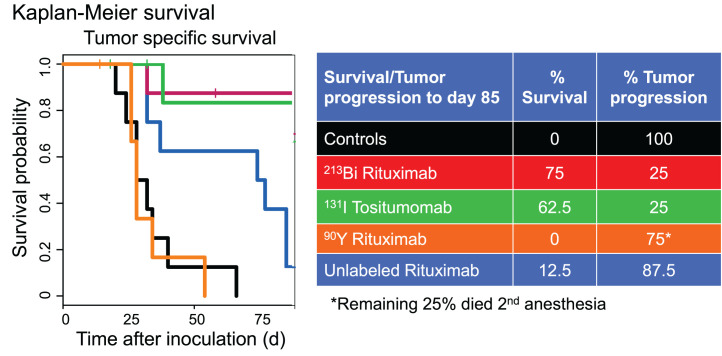
Experiment 4. Single treatment 4 d after injection of 10^6^ tumor cells intravenously (8 animals per group) was studied and survival assessed. Groups included controls (no treatment), 10 μg of unlabeled rituximab, 3,700 kBq of ^213^Bi-rituximab, 2,035 kBq of ^131^I-tositumomab, and 1,295 kBq of ^90^Y-rituximab. Cures were common with ^213^Bi-rituximab and ^131^I-tositumomab.

In experiment 4 (Figs. 5A–5D and 6), all untreated controls developed HLP at days 20–40. In the group treated with a 3,700-kBq dose of ^213^Bi-rituximab, only 25% of the mice had disease progression, and these died or developed HLP on days 32 and 135. In the group treated with 2,035 kBq of ^131^I-tositumomab, only 25% of the mice had disease progression, and these died or developed HLP on days 38 and 155. Of mice treated with a mass-equivalent dose of unlabeled rituximab, 87.5% had disease progression and died or developed HLP from days 32 to 37 or from days 74 to 85. All mice in the group receiving 925 kBq of ^90^Y-rituximab had disease progression and died or developed HLP from days 26 to 54.

Long-term remission or cure was defined as any animal without a significant increase in tumor signal above the pretreatment baseline on the final day of imaging in the 3 survival studies.

In experiment 2 (Figs. 3A and 3B), 1 of 4 (25%) animals were cured in the treatment groups given 2 doses of 10 μg of unlabeled rituximab; 2 of 6 (33%) animals given a single dose of 2,775 kBq of ^213^Bi-rituximab and 3 of 6 (50%) animals given 2 doses of 2,775 kBq of ^213^Bi-rituximab met criteria for long-term remission or cure. In the latter 2 groups, treated with 1 and 2 doses of the radiopharmaceutical, 1 mouse in each group died secondary to complications from anesthesia during imaging on day 29. These mice were tumor-free at the time of accidental death, without significant bioluminescent signal above baseline. All mice in each of these groups that ultimately progressed to death secondary to tumor had already developed significant bioluminescent signals by day 29. The 3 mice that progressed to death secondary to tumor in the single-dose group had 13.6, 1,308, and 1,508 times the absolute bioluminescent signal, on day 29, of the mouse that died prematurely from anesthesia. In the 2-dose group, the 2 mice that progressed had 51.7 and 60.5 times the absolute bioluminescent signal of the mouse that died prematurely in that group. These 2 animals would likely have been counted among the cured in their respective groups, except for their premature accidental deaths. Thus, a more accurate assessment of tumor cure rates could be considered as 50% in the group receiving a single dose of ^213^Bi-rituximab and 67% in the group receiving 2 doses of ^213^Bi-rituximab. None of the mice in the untreated control group or the group treated with 2 doses ^213Bi^-anti-HER2/*neu* were cured.

No cures were achieved in experiment 3 (Fig. 4; Supplemental Fig. 3). In experiment 4 (Figs. 5 and 6; Supplemental Figs. 4 and 5), animals were cured in treatment groups given 1 dose of 10 μg of nonradioactive rituximab, 1 dose of 3,700 kBq of ^213^Bi-rituximab, and 1 dose of 2,035 kBq of ^131^I-tositumomab; 1 of 8 (12.5%), 4 of 8 (50%), and 4 of 8 (50%) mice, respectively, met criteria for long-term remission or cure. None of the mice in the untreated control group or the group treated with ^90^Y-rituximab were cured. Two mice in the ^90^Y-rituximab group died prematurely secondary to complications of anesthesia on day 12. Both mice had tumor progression beyond baseline comparable to the other mice in the same group and are unlikely to have been cured. Two mice each in the ^213^Bi-rituximab and ^131^I-tositumomab groups died on days 58 and 89 and days 18 and 32, respectively, without any increase in tumor signal above baseline. Although these animals likely all represent additional cures, it is possible their premature death occurred as a result of treatment-related toxicity. Thus, these animals were likely cured; therefore, cure rates of 75% potentially can be claimed for both the group receiving 3,700 kBq of ^213^Bi-rituximab and the group receiving 2,035 kBq of ^131^I-tositumomab. BLI showed residual tumor in 1 animal each in the surviving groups treated with 3,700 kBq of ^213^Bi-rituximab and 2,035 kBq of ^131^I-tositumomab; these animals therefore were not cured.

Linear mixed-effects models were used compare rates of tumor progression among treatment groups. To assess tumor growth rates only among animals with actively growing tumor, animals that did not exhibit a significant increase in tumor signal above baseline pretreatment levels (cured animals and animals without significant tumor progression at the time of death secondary to complications of anesthesia or treatment-related toxicity) were removed from each group before this analysis. In all 4 in vivo experiments, the untreated control mouse groups had significantly higher rates of tumor progression than any other treatment or control group. In experiment 1, mice treated with a single dose of 1,295 kBq of ^213^Bi-rituximab progressed more slowly than mice treated with either 1,295 kBq of free ^213^Bi radiometal or 1,295 kBq of ^213^Bi-anti-HER2/*neu* (nonspecific antibody) (Fig. 2; Supplemental Table 1). In experiment 1, mice treated with a single dose of 3,700 kBq of ^213^Bi-rituximab progressed more slowly than those treated with a single dose of 1,295 kBq of ^213^Bi-rituximab.

In experiment 2, mice treated with 2 doses of 2,775 kBq of ^213^Bi-rituximab progressed more slowly than mice treated with 2 doses of 2,775 kBq of ^213^Bi-anti-HER2/*neu*. In experiment 2 (Figs. 3A and 3B), 2 doses of 2,775 kBq of ^213^Bi-rituximab given on days 7 and 13 resulted in slower tumor progression than 1 dose of 2,775 kBq of ^213^Bi-rituximab given on day 7. In series 3, animals were inoculated with twice the initial dose of tumor that those in series 2 received. All animals (*n* = 8 in each group) had disease progression and could be included in this analysis. In experiment 3, 2 or 3 doses of 2,775 kBq of ^213^Bi-rituximab given on days 7 and 12 or days 7, 12, and 19, respectively, significantly slowed tumor progression versus a single dose of 2,775 kBq of ^213^Bi-rituximab given on day 7 (*P* < 0.0001). The group treated with 2 doses had slower tumor progression than the group treated with 3 doses (*P* = 0.0229). Detailed statistical analyses of changes in BLI and of survival for experiment 4 are shown in Supplemental Tables 2–7 and Supplemental Figures 6, 7, 8A, 8B, and 9–11 (Supplemental Statistical Data Study 4), with BLI-change findings consistent with survival data.

Adverse events related to these experiments involved mortality from the anesthetic procedure (*n* = 6 distributed across all experiments). Histopathology was performed on multiple organs from representative animals across experiments. Untreated animals had a high tumor burden in multiple organs, including the brain, marrow, kidney, liver, and mesentery. A 1,295- or 3,700-kBq dose of ^213^Bi-rituximab markedly reduced tumor burden. Mice treated with 1,295 or 3,700 kBq of ^213^Bi-rituximab had either no tumor cells in any organs or very limited residual tumor cells at a single site. Spleens from mice receiving 1,295 or 3,700 kBq of ^213^Bi-rituximab were visibly smaller and had mild lymphoid depletion. There was no other evidence of significant toxicity in any of the organs examined histologically, including kidneys.

## DISCUSSION

Our studies have demonstrated several important findings: ^213^Bi-rituximab could reliably be produced. In vitro CD20-positive specific lymphoma cell killing was achieved. The intravenous tumor model system yielded disseminated NHL with a predictable pattern of lethality, including development of hind limb paralysis. ^213^Bi-rituximab was more effective at tumor cell kill in vitro and in vivo than free ^213^Bi or ^213^Bi-anti-HER2/*neu* (nonspecific antibody). A single dose of 3,700 kBq of ^213^Bi-rituximab given 6 d after tumor injection was more active than 1,295 kBq of ^213^Bi-rituximab or unlabeled rituximab. Dose–response relationships were identified.

Although tumor growth delays could be achieved, animals with high tumor burdens at the start of therapy could not reliably be cured by ^213^Bi-rituximab, even with repeated dosing. For lower injected tumor cellular doses, including animals treated earlier in the course of their disease, cures were common, particularly with ^213^Bi-rituximab. In experiment 4, in which treatment was initiated 4 d after 1 million cells were injected, 1 dose of 3,700 kBq of ^213^Bi-rituximab and 1 dose of 2,035 kBq of ^131^I-tositumomab achieved cures in 4 of 8 (50%) and 4 of 8 (50%) mice, respectively, meeting the criteria for long-term remission or cure, whereas 2 animals each in the ^213^Bi-rituximab and ^131^I-tositumomab groups died without detectable tumor; thus, a 75% cure rate could be considered. None of the mice in the untreated control group or the group treated with ^90^Y-rituximab were cured.

α-emitters have previously been used to radiolabel anti-CD20 antibodies. Roscher et al. showed activity of ^213^Bi-rituximab in vitro in chemosensitive and chemoresistant lymphoma cells. ^213^Bi-rituximab treatment appeared to restore caspase activity in vitro (*21*). Vandenbulcke et al. have shown a relative biological effectiveness of up to 5 for ^213^Bi-rituximab in vitro in killing human chronic lymphocytic leukemia cells (*13*). Intact ^213^Bi-rituximab was also active in vitro in our experiments, in which radioantibody access to tumor cells was nearly immediate, paralleling the antibody access to tumor cells in our in vivo model.

^213^Bi-1F5 intact anti-CD20 was evaluated for biodistribution to subcutaneous lymphoma xenografts by Park et al. (*12*). At 45 min after injection, the tumor-to-blood uptake ratio was only 0.06, with 3 percentage injected dose per gram in tumor, equivalent to a nonspecific antibody. Given this poor targeting to subcutaneous tumor models, investigators did not perform therapy studies with the ^213^Bi-labeled intact anti-CD20, rather focusing on a pretargeting approach (*12*).

We showed that the intravenous tumor model is treated successfully by the intravenous ^213^Bi-rituximab, with cures possible in many animals (series 4 experiments). Thus, intact antibodies that have very slow localization to subcutaneous tumors have substantial therapeutic efficacy, even with the 46-min t½ of ^213^Bi, in the setting of disseminated lymphoma. Ostensibly, this finding defies the common wisdom regarding the suitable t½ for therapeutic isotopes used with intact monoclonal antibodies. Our data show the clear feasibility of intact radioantibody therapy with short-lived α-emitters in systems in which intact antibodies reach tumor quickly. Schmidt et al. have used ^213^Bi-anti-CD20 antibodies to treat 12 patients with NHL in an early-phase study (*22*).

^211^At-IF5 (anti-CD20 antibody) slowed tumor growth in subcutaneous lymphoma tumor xenografts but did not achieve cures. However, an approximately 70% cure rate was seen with a solitary injection of ^211^At-IF5 2–6 d after an intravenous injection of 1 million tumor cells with stem cell support (*23*). Their cure rate is similar to ours using a single 2,775-kBq dose of ^213^Bi-rituximab, but we did not use stem cell support. α-autoradiography of the subcutaneous tumors in a study by Green et al. showed heterogeneous dose delivery, likely accounting for the lack of cures in the subcutaneous tumors (*23*). Higher tumor burdens in animal models are also associated with faster antibody clearance from the bloodstream, potentially limiting treatment efficacy (*24*). α-emitting ^212^Pb (t½, 10.6 h)-rituximab has demonstrated antitumor efficacy in a B-cell lymphoma model in which 25,000 tumor cells were injected and treatment was initiated 11 or more days after injection (*25*).

^227^Th (t½, 18.7 d)-rituximab was used to treat NHL xenografts (not minimal residual disease), with some cures (*26**,**27*). ^227^Th-rituximab was superior in treatment efficacy in vivo to ^90^Y-ibritumomab tiuxetan and had a higher relative biological effectiveness (*26*). ^227^Th decays to ^223^Ra, which can travel to normal bone, a potential limitation.

^213^Bi-rituximab, even with its very short t½, has a significant therapeutic advantage over the first-generation ^90^Y-anti-CD20 therapy, in a disseminated minimal residual disease model. Our data are intriguing as well, in that a high cure rate was seen when a single dose of ^131^I-tositumomab was used in the animal model, with therapeutic efficacy comparable to ^213^Bi-rituximab. It appears that the low-energy ^131^I β-emission, coupled with the 8-d t½ of ^131^I (to allow continued irradiation of tumors over days), is a viable choice for eliminating minimal residual disease despite the limitation of the longer β-pathlength. These preclinical observations may help explain some of the very long clinical remissions reported using ^131^I-tositumomab (*28*). Because we had a very limited supply of ^131^I-tositumomab, it was not possible to systematically compare the β-emitting therapy with ^213^Bi-rituximab in larger studies. It is unlikely that the difference in the therapeutic effects was due to the differences in the underlying anti-CD20 antibodies, with rituximab being a mouse–human chimera and tositumomab a purely murine reagent.

Our studies add to the emerging literature showing that a wide range of α-emitters can be used to treat human malignancies in vivo in animal models, and our findings suggest considerable potential for in vivo translation. The short t½ of ^213^Bi, 46 min, is a practical logistic limitation but allows for a very high dose rate if the α-emitter binds quickly to the tumor. Longer-lived α-emitters may be of greater potential. ^225^Ac (whose 10-d t½ may be ideally matched to the localization kinetics of intact rituximab or related antibodies without the ^223^Ra daughter of ^227^Th, though with a ^213^Bi daughter that can circulate distantly.

It remains unclear why we were unable to achieve cures in all cases. A logical presumption is that the larger tumor burden with more bulky disease limited ^213^Bi-rituximab accessibility. This was not problematic when therapies were started earlier, such as in our fourth experiment, with therapy given 4 d after tumor cell injection. Our histology studies showed the emergence of bulky tumor, to which good access of the radioantibodies during the t½ of the ^213^Bi-rituximab treatment would not have been likely.

There are limitations to our study: β-emitter–labeled anti-CD20 antibodies were limited to the final study, and ^131^I-tositumomab (a purely murine monoclonal antibody) was compared with ^90^Y and ^213^Bi-rituximab (a mouse–human chimeric antibody). Thus, there could have been varying impacts of the antibody itself. Although unlabeled rituximab has activity in our system, it is unlikely that the excellent results with ^131^I-tositumomab are due simply to the murine antibody (*3*). It is also not totally clear that the maximum tolerated dose was given for each radioimmunotherapeutic agent; thus, there is room for additional study.

## CONCLUSION

Anti-CD20 radioimmunotherapies have not been a great commercial success to date, but they are very active therapies, and a variety of B-cell lymphomas remain incurable. The efficacy of the short-lived ^213^Bi-rituximab in curing disseminated NHL in animals lends support to the reevaluation of anti-CD20 radioimmunotherapy with α-emitter labeling, potentially using the longer-lived ^225^Ac.

## DISCLOSURE

Financial support was provided through P50CA096888. No other potential conflict of interest relevant to this article was reported.

## Notes

KEY POINTS **QUESTION:** Can ^213^Bi-anti-CD20 rituximab be produced and effectively treat micrometastatic human NHL in vitro and in an animal model? **PERTINENT FINDINGS:** ^213^Bi-rituximab showed specific lymphoma cytotoxicity in vitro and significant antilymphoma treatment efficacy in vivo versus free ^213^Bi, ^213^Bi control antibody, ^90^Y-rituximab, and rituximab. In the group treated with 3,700 kBq of ^213^Bi-rituximab or ^131^I-tositumomab, 75% of the mice became tumor-free, with cures being most common. **IMPLICATIONS FOR PATIENT CARE:** ^213^Bi-rituximab is an active therapeutic for NHL, although a longer-lived α-emitter would likely be more effective.
